# Metabolic engineering of *Thermoanaerobacterium aotearoense* strain SCUT27 for biofuels production from sucrose and molasses

**DOI:** 10.1186/s13068-023-02402-3

**Published:** 2023-10-21

**Authors:** Kaiqun Dai, Chunyun Qu, Jun Feng, Yang Lan, Hongxin Fu, Jufang Wang

**Affiliations:** 1 School of Biology and Biological Engineering, South China University of Technology, Guangzhou, 510006 China; 2 College of Light Industry and Food Science, Guangdong Provincial Key Laboratory of Science and Technology of Lingnan Special Food Science and Technology, Zhongkai University of Agriculture and Engineering, Guangzhou, 510225 China; 3 Guangdong Provincial Key Laboratory of Fermentation and Enzyme Engineering, South China University of Technology, Guangzhou, 510006 China

**Keywords:** *Thermoanaerobacterium*, Sucrose, Metabolic engineering, Cane molasses, Biofuels

## Abstract

**Background:**

Sucrose-rich sugarcane trash surpasses 28 million tons globally per year. Effective biorefinery systems could convert these biomasses to bioproducts, such as bioethanol from sugarcane sucrose in Brazil. Thermophilic microbes for biofuels have attracted great attention due to their higher fermentation temperature and wide substrate spectrum. However, few thermophiles using sucrose or molasses for biofuels production was reported. *Thermoanaerobacterium aotearoense* SCUT27 has been considered as an efficient ethanol producer, but it cannot directly utilize sucrose. In this study, various sucrose metabolic pathways were introduced and analyzed in Thermoanaerobaterium.

**Results:**

The sucrose-6-phosphate hydrolase (*scrB*), which was from a screened strain *Thermoanaerobacterium thermosaccharolyticum* G3-1 was overexpressed in *T. aotearoense* SCUT27 and endowed this strain with the ability to utilize sucrose. In addition, overexpression of the sucrose-specific PTS system (*scrA*) from *Clostridium acetobutylicum* accelerated the sucrose transport. To strengthen the alcohols production and substrates metabolism, the redox-sensing transcriptional repressor (*rex*) in *T. aotearoense* was further knocked out. Moreover, with the gene arginine repressor (*argR*) deleted, the ethanologenic mutant P8S10 showed great inhibitors-tolerance and finally accumulated ~ 34 g/L ethanol (a yield of 0.39 g/g sugars) from pretreated cane molasses in 5 L tank by fed-batch fermentation. When introducing butanol synthetic pathway, 3.22 g/L butanol was produced by P8SB4 with a yield of 0.44 g alcohols/g sugars at 50℃. This study demonstrated the potential application of *T. aotearoense* SCUT27 for ethanol and butanol production from low cost cane molasses.

**Conclusions:**

Our work provided strategies for sucrose utilization in thermophiles and improved biofuels production as well as stress tolerances of *T. aotearoense* SCUT27, demonstrating the potential application of the strain for cost-effective biofuels production from sucrose-based feedstocks.

**Supplementary Information:**

The online version contains supplementary material available at 10.1186/s13068-023-02402-3.

## Introduction

Currently, the main energy source in the world is unsustainable fossil fuel, whereas the high consumption of it has a huge impact on the environment, such as global warming, and the availability of fossil fuels could be seriously influenced by economics and politics [1]. Thus, cheap biomass as renewable energy resources has been used for biofuels production to address the energy demands for the future [2]. The first-generation (1G) bioethanol produced, from sugarcane, beet and starch (primarily from corn and wheat grain), still cannot be fully substituted by the second-generation (2G) biofuels from lignocellulosic biomass so far [3]. Currently bioethanol is mainly from sugarcane sucrose in Brazil and from corn starch in the United States, which together account for ∼ 85% of a global yearly production [4]. In addition, ∼ 80% of the microorganism used for ethanol production in North America are genetically engineered strains, including those expressing amylases and engineered to gain higher yields [4]. The potential of sugarcane as the substrate to replace traditional biomass for ethanol production was evaluated to be more economically feasible in Southern Africa and Mexico [5, 6]. The molasses as a by-product of sucrose production and a non-food feedstock mainly composed of sucrose, glucose and fructose, is one of the most important and suitable substrates for production of biofuels and other bioprocess-based commodities [1, 7]. Swaziland may increase power production by 40% (from bagasse) and replace 60% of cooking fuel or 30% of liquid fossil fuel by utilizing molasses for ethanol production [5].

The functional genes for sucrose utilization had been introduced to several microorganisms for efficient sucrose catabolism. In mesophiles, Jeong et al. introduced β-fructofuranosidase from *Mannheimia succiniciproducens* to *Escherichia coli* K-12 for L-threonine production using sucrose [8]. Park et al. established a sucrose metabolic pathway in *Ralstonia* by over-expressing the gene *sac* from *M. succiniciproducens* for poly(3hydroxybutyrate) and poly(3-hydroxybutyrateco-lactate) production from sucrose [9]. *C. acetobutylicum* can use sucrose naturally and recently it was used for the production of biobutanol from black strap molasses [10]. *C. tyrobutyricum* can efficiently metabolize sucrose and even untreated cane molasses for butyrate production after *scrBAK* over-expression from *C. acetobutylicum* [11, 12]. Zhang et al. co-expressed an aldehyde/alcohol dehydrogenase gene along with *scrBAK* from *C. acetobutylicum* in *C. tyrobutyricum* (Δ*ack*), and the resultant mutant could produce butanol from sucrose and sugarcane juice [12]. Other products from molasses or sugarcane wastes were also reported, such as L-lysine, poly (L-malic acid), polyhydroxyalkanoates and so on [7, 13].

The optimal growth temperature of thermophilic anaerobes is higher than 50 °C, which showed tremendous advantage on lignocellulose degradation and reaction kinetics improvement [14]. A higher culture temperature can lower the cooling costs as well as the risk of microbial contamination [15]. In addition, enzymes derived from thermophiles are generally thermostable and resistant to the conditions present in industrial manufacturing, such as high salt or solvent concentrations [16–18]. Therefore, it is essential to develop thermophiles as versatile hosts using various carbon sources especially sucrose, which is one of the most abundant and relatively cheap carbon sources from sugarcane, sugar beet and molasses, for efficient production of target products by inherent or heterologous metabolic engineering.

*T. aotearoense* strain SCUT27, isolated from hot spring in South China, can efficiently produce ethanol at 55 °C with a broad substrate spectrum, including xylan, glucose, cellobiose, fructose, xylose, mannose, galactose, arabinose and so on [19–21]. However, *T. aotearoense* SCUT27 cannot use sucrose, even though *scrBAK* from *C. acetobutylicum* was overexpressed in this study. Here, a new *T. thermosaccharolyticum* strain G3-1 was first screened and identified to be capable to metabolize sucrose through expressing thermostable enzyme ScrB. Then, *T. aotearoense* SCUT27 was engineered to produce ethanol and butanol from sucrose by introducing a series of heterogenous sucrose catabolism and butanol production pathways. In addition, the global transcriptional regulators *rex* and *argR* which were previously demonstrated to function as global transcription factors for alcohols accumulation and inhibitors tolerance were genetically edited for ethanol and butanol production. Using the engineered thermophilic bacteria and pretreated molasses, biofuels or other biochemicals could be produced with great economic advantages.

## Results and discussion

### Isolation and identification of a sucrose-utilization strain *T. thermosaccharolyticum* G3-1

Some isolates with mucoid colonies from a spring in Yunnan province were screened out as thermophilic sucrose consumers on MTC (medium for thermophilic clostridia) agar plates with sucrose as the sole carbon source. After 16 s rDNA sequencing, it showed that all of them were categorized as one of the *T. thermosaccharolyticum* species, named as strain G3-1. As shown in Additional file 1: Fig. S1A, *T. thermosaccharolyticum* G3-1 is most closely related to *T. thermosaccharolyticum* DSM571 (NCBI Accession Number CP002171.1), whose genome has the operon of sucrose metabolism.

However, the growth rate of *T. thermosaccharolyticum* G3-1 was much slower compared with *T. aotearoense* strain SCUT27, especially under glucose and fructose, and the metabolic rate of sucrose in strain G3-1 was obviously slower than that of glucose or fructose (Additional file 1: Fig. S1B). Possibly, the genes of sucrose catabolism operon in *T. thermosaccharolyticum* G3-1 were regulated by the potential carbon catabolite repression (CCR) when glucose or other advantageous carbon source existed, as the transcriptional repressor (*scrR*) or anti-terminator gene (*scrT*) functioned in other strains [22]. In addition, the products in the aqueous phases of strain G3-1 included lactate, butyrate, acetate and ethanol, more complicated than those in *T. aotearoense* SCUT27 (Additional file 1: Fig. S1B, right), thus the possibility of further utilization is reduced, especially under the condition without mature genetic modification technology. All above indicated *T. thermosaccharolyticum* G3-1 may not be suitable as an industrial biofuel producer, while *T. aotearoense* SCUT27, which has a great fermentation performance [23] and a mature platform for metabolic engineering [24], seemed to have more potential as a promising candidate for sucrose utilization through metabolic engineering for biofuels production.

### Engineering of *T. aotearoense* SCUT27 for sucrose utilization

**Fig. 1:**
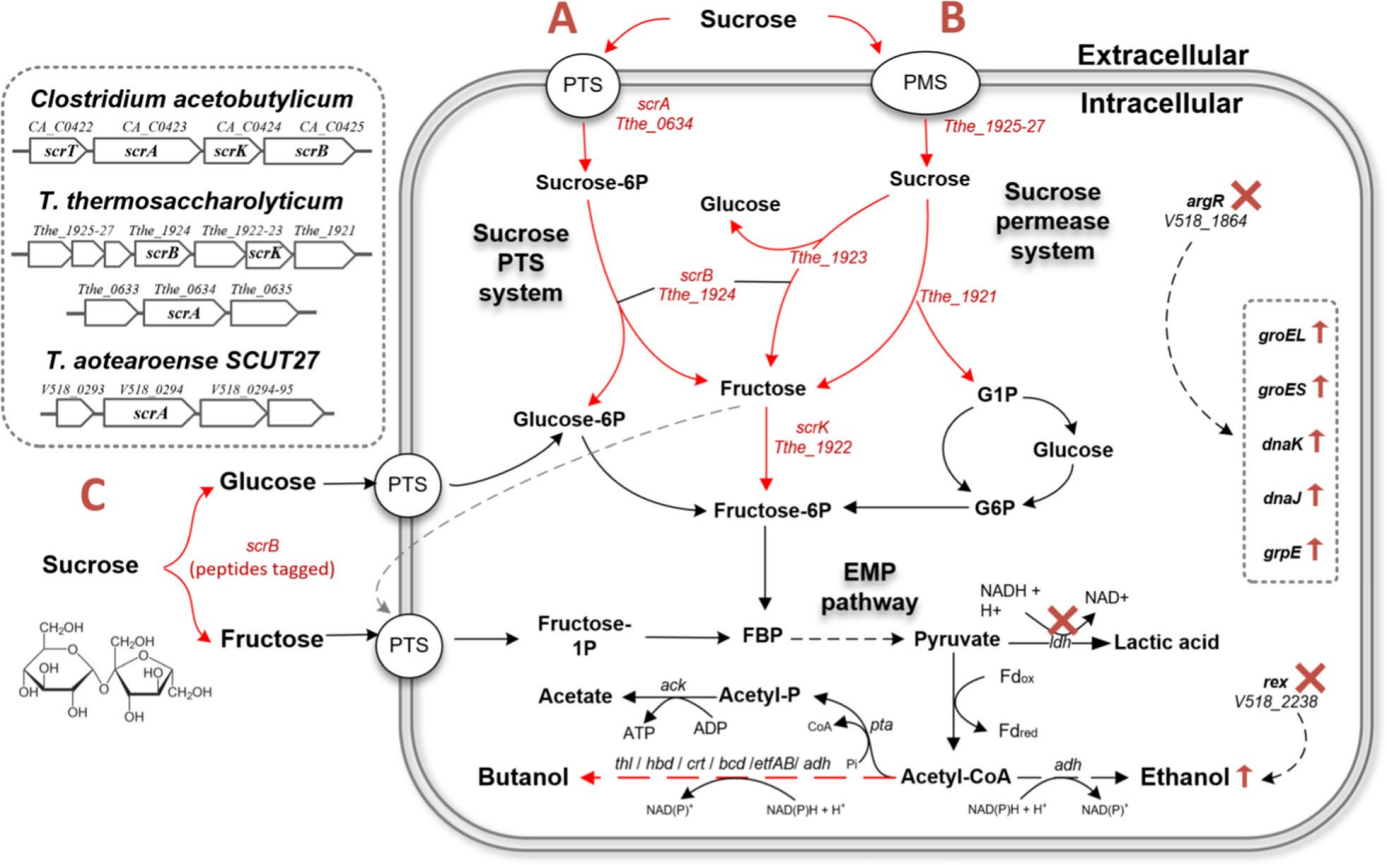
Schematic representation of the sucrose utilization pathway and metabolic engineering strategies used in strain SCUT27. **A** Sucrose PTS system, **B** sucrose permease system, and **C** secreted sucrose 6-phosphate hydrolase system. Solid lines indicate metabolic pathway (the heterogenous are in red) or phosphoryl group transfer. Red cross represents the gens deletion and red arrows show the upregulated expression of the genes. The source of sucrose-utilizing genes was shown in left. G1P, glucose 1-phosphate; G6P, glucose 6-phosphate; FBP, fructose 1,6-bisphosphate; PMS, permease system

Sugars can be transported into the cell via various transporters, such as phosphoenolpyruvate (PEP)-dependent phosphotransferase system (PTS), ATP-binding cassette (ABC) transporter, permeases or proton symporter [8, 11, 25]. In most Clostridia (including Thermoanaerobacterium) such as *C. acetobutylicum*, PTS-dependent transporter ScrA (CA_C0423, Fig. 1), sucrose-6-phosphate hydrolase or ScrB (CA_C0425) and fructokinase ScrK (CA_C0424) are responsible for sucrose transport and metabolism [26–28]. First, *scrBAK* from *C. acetobutylicum* was introduced into *T. aotearoense* SCUT27 and resultant mutant P8S01, unfortunately, could not grow in sucrose, possibly due to inactivation of mesothermal enzyme in higher temperature. In *T. thermosaccharolyticum* DSM571, the potential sucrose-metabolic genes *scrB*, *scrA* and *scrK* were matched through BLAST with those in *C. acetobutylicum* ATCC 824 (Fig. 1, left). Those three proteins had 36.71%, 35.19% and 28.80% amino acid sequence identity, respectively. Potential ScrA (V518_0294), responsible for sucrose transport, also existed in *T. aotearoense* SCUT27 with 32.09% amino acid sequence identity. In addition, sucrose-permease system (Tthe_1925-27) were identified in the specific sucrose-metabolism operon of *T. thermosaccharolyticum* DSM571 (Fig. 1).

**Fig. 2:**
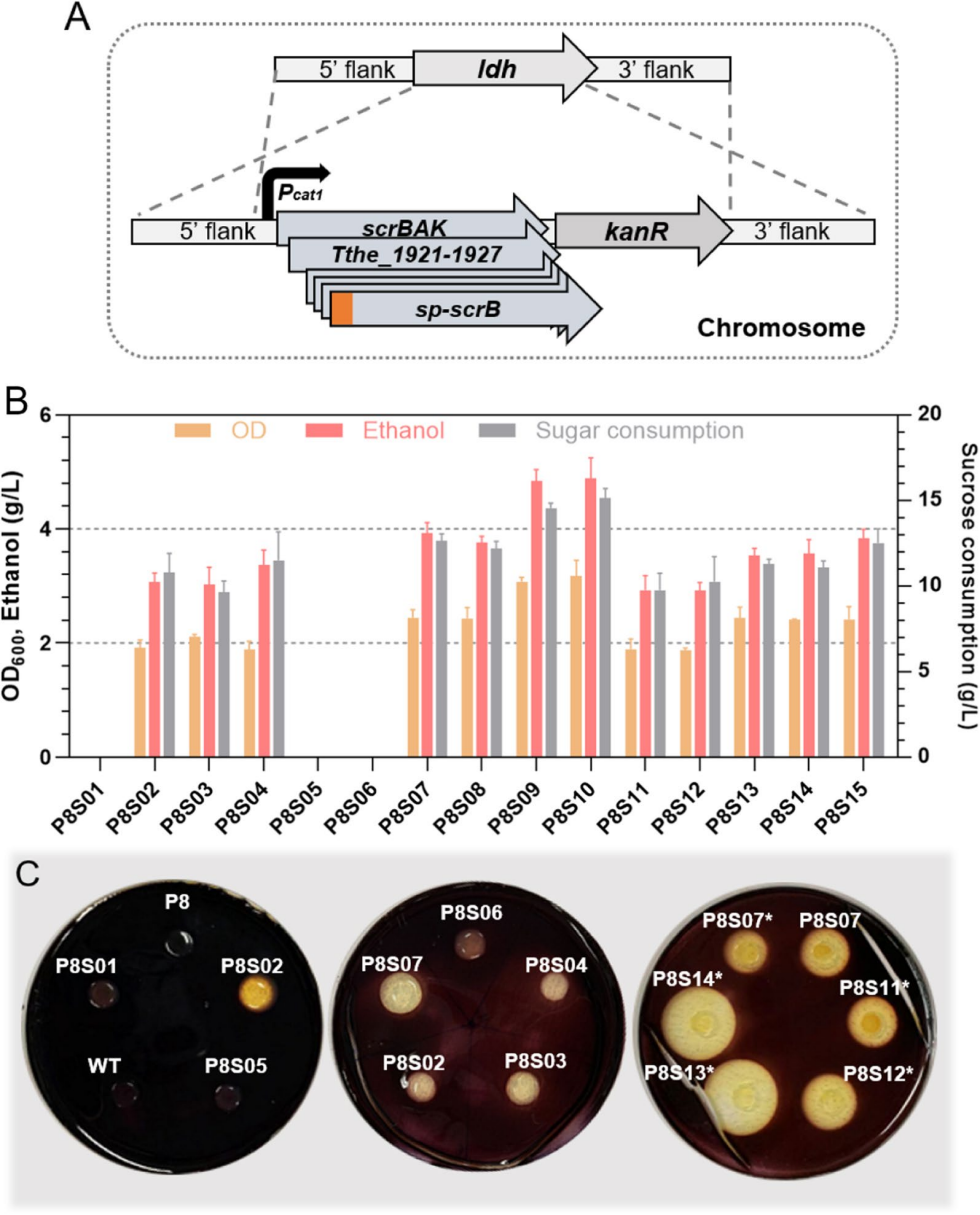
Various engineered strains and their traits. **A** Schematic diagram of the engineering for introducing sucrose metabolic pathways. **B** Maximum OD, sugar consumption and ethanol production for different engineered strains under 20 g/L sucrose in 30 h. **C** Enzyme activity test with the cell lysis buffer, or with the liquid supernatant of cell culture medium without cell lysis (marked with “*”). Presented data are derived from three independent experiments

All genes above were successfully amplified by PCR from the gDNA of *T. thermosaccharolyticum* G3-1 (The gene locus number of *T. thermosaccharolyticum* DSM571 was used as that of *T. thermosaccharolyticum* G3-1 in this paper), and the sequencing results showed that the genes had highly conserved coding regions compared with those of *T. thermosaccharolyticum* DSM571 (Additional file 1: Table S1). For stable inheritance, all heterologous genes were expressed on the genome of *T. aotearoense* SCUT27. The gene site of lactate dehydrogenase (*ldh*) responsible for lactic acid production was chosen for replacement with introduced genes via homologous recombination (Fig. 2A), since the deletion of *ldh* not only eliminated the byproduct lactate, but also improved the ethanol production and cell growth [20]. The genes *scrB* and *scrA* from *T. thermosaccharolyticum* G3-1 with the strong promoter P_*cat1*_ were first overexpressed in *T. aotearoense* SCUT27, and as expected, the mutant P8S02 could grow under sucrose as sole carbon resource (Fig. 2B). However, compared with the mutant P8S03 with ScrB overexpression only, additional overexpression of *scrA* did not improve the sucrose utilization, which could probably be attributed to the fact that *T. aotearoense* SCUT27 has potential native transporter ScrA (V518_0294) for sucrose transport.

In addition, the whole operon of sucrose metabolism (Tthe_1921-27) from *T. thermosaccharolyticum* G3-1 containing the potential sucrose permeases and genes for sucrose degradation (Fig. 1) was directly cloned into *T. aotearoense* SCUT27, to improve sucrose consumption. The resultant strain P8S04 could grow on sucrose, while the growth rate was similar with P8S02 (Fig. 2B). We hypothesized that a single promoter for several genes might lead to weak expression of the later effective genes, thus ignoring the presence of some useful genes. The gene Tthe_1921 is annotated as sucrose phosphorylase (EC 2.4.1.7), which could degrade sucrose to α-D-glucose-1P and fructose [29]. In addition, Tthe_1923 is annotated as a kind of oligo-1,6-glucosidase [EC:3.2.1.10] with the ability of catabolizing sucrose to glucose and fructose. However, expression of the genes each alone with P_*cat1*_ in *T. aotearoense* SCUT27 still did not show obvious sucrose utilization (Fig. 2B). Based on above results, it was concluded that *scrB* was the key gene in the cluster of sucrose catabolism and permeases did not function correctly in *T. aotearoense* SCUT27, the reason needs to be further explored. It should be noted that similar fermentation results under glucose were observed for the engineered strains (P8S01 to P8S06), indicating that expression of these heterogenous genes did not show significant metabolic burden on *T. aotearoense* SCUT27, whereas the growth rate of the strains (P8S02 to P8S04) in sucrose was slower than that in glucose (data not shown).

In sucrose PTS system (Fig. 1A), the sucrose absorbed by the specific PTS transporters comes into the cytoplasm as sucrose-6-phosphate and then is degraded to glucose-6-phosphate and fructose, which are further converted to fructose-6-phosphate under related enzymes, finally flowing into EMP pathway. However, no native ScrK (this enzyme phosphorylates intracellular fructose) was found by BLAST in *T. aotearoense* SCUT27, indicating the fructose in cytoplasm may not be directly used. In some bacteria, such as *Corynebacterium glutamicum*, the fructose in cytoplasm would be delivered outside the cells and transported into cells again via fructose PTS system to yield intracellular fructose 1-phosphate [30, 31]. The PTS system is an energy-consumed process and inevitable energy competition exists among various PTS carbon sources [32]. Thus, the re-transport and low metabolic rate of fructose (compared with glucose in *T. aotearoense* SCUT27, data not shown) probably account for slow growth of recombinant SCUT27 strains with sucrose. The same phenomenon may also exist in permease system (Fig. 1B), as the sucrose was transported into cell and then catabolized to unphosphorylated fructose and glucose or glucose-1-P.

For further accelerating sucrose assimilation, the genes *scrK* (The_1922) from *T. thermosaccharolyticum* G3-1 and *scrAK* (CA_C0423-0424) from *C. acetobutylicum* were also expressed based on the mutant P8S03, obtaining the strains P8S07 and P8S08. Interestingly, as shown in Fig. 2B, the engineered strain P8S07 containing the gene *scrA*(CA) displayed an obvious improvement on sucrose utilization, while the strain P8S08 with overexpression of genes *scrK* (CA and G3-1) showed no significant change. The sugar consumption and ethanol production of strain P8S07 were increased by 31.2% and 30.2%, respectively, compared with those of strain P8S03, showing that ScrA from *C. acetobutylicum* was functional in *T. aotearoense* SCUT27.

### Constructing secretory expression of ScrB by signal peptide tagging

As we speculated that one key obstacle for sucrose metabolism was its transportation, β-fructofuranosidase system reported by Lee [8] was attempted in this study to facilitate hydrolysis of sucrose outside the cell (Fig. 1C). The excreted β-fructofuranosidase (e.g., SacC from *M. succiniciproducens*, 27.1% amino acid sequence identities with ScrB of *T. thermosaccharolyticum* G3-1) in the culture medium could hydrolyze sucrose directly to glucose and fructose, then those two monosaccharides were further transported into cell by their respective PTS systems, which is an energy efficient process for carbon source utilization (excreted β-fructofuranosidase, sucrose PTS, and sucrose permease system consume 1, 1.5, and 2 mol of ATP to produce each mole FBP, respectively) and resulted in an increased pyruvate pool for ethanol production [8]. *T. aotearoense* SCUT27 could co-metabolize glucose and fructose with no CCR (data not shown), and thus may consume sucrose rapidly if sucrose-6-phosphate hydrolase could secret outside cells efficiently with assistance of signal peptides [33, 34].

Whether ScrB of *T. thermosaccharolyticum* G3-1 was a secretory enzyme was first analyzed by prediction tools, and the results showed no significant signal peptide was predicted in *scrB* by SignalP-5.0 [35], while transmembrane helices was captured by DeepTMHMM [36]. Four putative signal peptides predicted by SignalP-5.0 from the annotated genome of *T. aotearoense* SCUT27 (Additional file 1: Table S2) were added in the N-terminus of the ScrB, respectively. To identify the ScrB location and activity toward sucrose, the hydrolysis circle size of supernatant or cell lysates of the engineered strains on sucrose plates was measured, as well as the concentrations of glucose/fructose in medium containing sucrose with 10% of the supernatant or cell lysates of different mutants’ culture. As shown in Fig. 2C and Additional file 1: Table S3, the supernatant of P8S13 and P8S14 (tagged with the signal peptides of Sec/SPI) showed the obvious sucrose degradation, while strains without signal peptide (P8S07, P8S04, etc.) or P8S11–12 with the signal peptides of Sec/SPII had no or lower sucrose degradation, indicating ScrB with Sec/SPI signal peptide tagging had a great secretion effect. In addition, the secreted ScrB (supernatant of strains P8S13–14) degraded sucrose faster than that of intracellular ScrB (cell lysates of P8S07), since the hydrolysis circles on sucrose plates formed with bigger size and the concentrations of glucose and fructose in medium were higher (Fig. 2C; Additional file 1: Table S3). However, the growth and fermentation results of the mutants with the signal peptides were not superior to the previous engineered strain P8S07 (Fig. 2B). It seemed that the rate of extracellular sucrose hydrolyzing with monosaccharides transporting was equal to that of sucrose transporting and subsequent intracellular catabolism in *T. aotearoense* SCUT27. On the other hand, the* K*_m_ value of ScrB here toward extracellular sucrose and intracellular sucrose-6-phosphate may be quite different, as the *K*_m_ for sucrose of *C. glutamicum* ScrB (26.8% amino acid sequence identities with ScrB of *T. thermosaccharolyticum*) is 190 Mm, while *K*_m_ for sucrose-6-P is only 0.04 mM [37]. All in all, although the resultant strains with peptide-tagged scrK such as P8S13 had a stronger ability to hydrolyze extracellular sucrose, the engineered strain P8S07 still had better growth on sucrose than strain P8S13, attributing to the efficiency of sucrose PTS transport and/or sucrose-6-P metabolism.

When combining intra- and extracellular catabolism of sucrose by overexpressing sp4–scrB in P8S07, no significant improvement of growth rate was observed in strain P8S15 (Fig. 2B). Thus, the hydrolase activity of ScrB was the main limitation in sucrose metabolic system, as the lower enzyme activity led to less biomass and products under sucrose than that under glucose. On the other hand, the disturbance of intracellular reducing equivalents may account for the growth difference under various substrates. When the products of *C. glutamicum* depending on NADPH, the yield of products on sucrose or fructose was lower than that on glucose, due to a reduced flux through the pentose phosphate pathway [38], as here the bioethanol produced in strain SCUT27 also relied on NAD(P)H [39].

### Engineering of strain SCUT27 for butanol production from sucrose

**Fig. 3:**
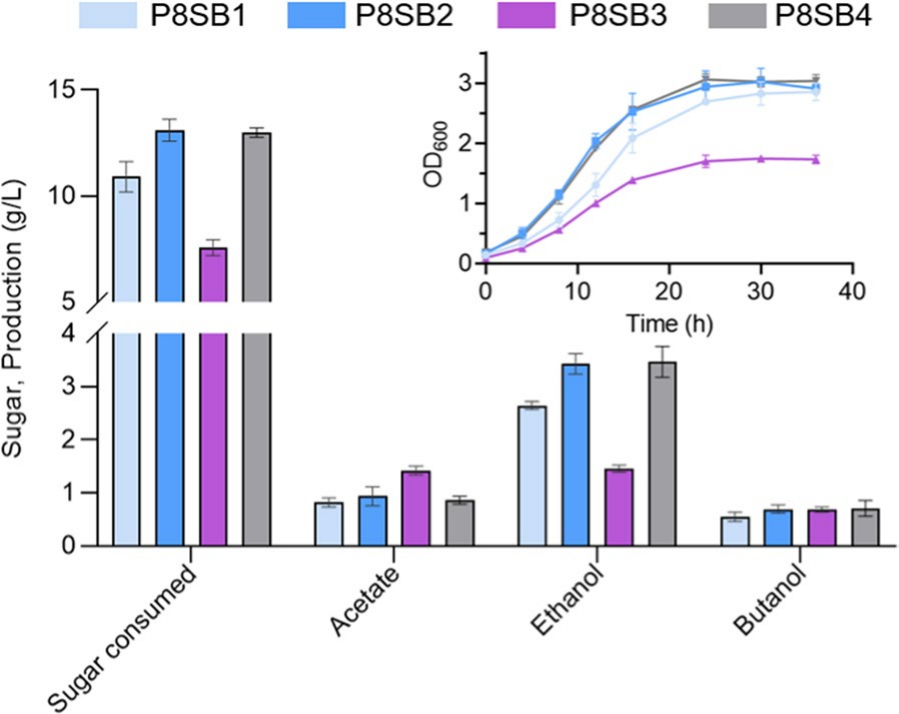
Fermentation profiles of the engineered strains for butanol production with ~ 20 g/L sucrose

Although the butanol biosynthetic pathway was present in *T. thermosaccharolyticum*, most strains including *T. thermosaccharolyticum* G3-1 produced little butanol, possibly due to the low enzyme activity for butyryl-CoA reduction to butanol [40]. In addition to aldehyde/alcohol dehydrogenases, the key genes for butanol production include *thl*, *hbd*, *crt*, *bcd*, *etfA* and *etfB* expressing enzymes thiolase, β-hydroxybutyryl CoA dehydrogenase, crotonase, butyryl CoA dehydrogenase, and electron transfer flavoproteins subunit A and B, respectively [41]. In this study, the operon *crt-bcd-etfAB-hbd-thl* from *T. thermosaccharolyticum* G3-1 as well as gene *adhE2* from *C. acetobutylicum* were cloned into the chromosome of *T. aotearoense* SCUT27 for butanol production from sucrose via an ethanol–butanol (EB) fermentation mode instead of the traditional ABE fermentation. The resultant strain P8SB1 successfully produced butanol but more ethanol with a ratio of butanol/ethanol ~ 1:4.8 (Fig. 3).

Moreover, in addition to *ldh* deletion, *rex* (redox-sensing transcriptional repressor) which senses the intracellular redox state and controls the metabolic pathway to maintain redox homeostasis was also deleted for enhanced alcohol production in *T. aotearoense* SCUT27 as our previous work [19]. In addition to the Rex binding sites of *adhE* and *adhA* had been reported by Qu [19], and a potential site was found in the promoter region of *crt* (Additional file 1: Fig. S2A) which was also reported in *C. acetobutylicum* [42]. Deletion of Rex could prevent the expression of downstream genes at these sites from being inhibited by high concentrations of NADH [43]. As shown in Fig. 3, compared with mutant P8SB1, the mutant P8SB2 (*rex* deleted) produced more ethanol (1.29-fold) and butanol (1.26-fold), with a yield of 0.26 and 0.05 g/g sucrose at 55℃, respectively, which was not only attributed to the increased ADHs expression and intracellular reducing power as reported by Qu [19], but also the enhanced *crt* expression (Additional file 1: Fig. S2B).

As the native *adhE* is specialized for the synthesis of acetaldehyde and ethanol in *Thermoanaerobaterium,* while *adhE2* is mainly used for the synthesis of butyraldehyde and butanol in *C. acetobutylicum* [39, 44], *adhE* of *T. aotearoense* SCUT27 was further deleted, aiming to direct more carbon and electron flux from ethanol toward butanol. The result showed that the ratio of butanol/ethanol was improved to 1:2.1 in the mutant P8SB3; however, its growth rate and alcohols production was low (Fig. 3). It suggested that the native AdhE in *T. aotearoense* SCUT27 was necessary for strain growth (*adhE* deletion in wild type also led to the growth inhibition of *T. aotearoense* SCUT27 as shown in Additional file 1: Fig. S3), and replacing native *adhE* with *adhE2* seemed hardly to eliminate the effect on growth of *T. aotearoense* SCUT27. The ethanol produced by P8SB3 was possibly catalyzed by AdhE2 from *C. acetobutylicum* or other ADHs in *T. aotearoense* SCUT27, such as AdhA [39, 45].

In addition, it was reported that low temperature was beneficial for alcohol production [22, 46, 47] as well as the activity of AdhE2 from mesophilic bacteria. The results showed the change of temperature affected the growth of the strain P8SB2 and the alcohols distribution (ratio of ethanol/butanol increased to ~ 1:3.3 with less ethanol and more butanol production at 50℃ compared to that at 55 ℃) (Additional file 1: Fig. S4). Furthermore, the mutant P8SB2 at 50℃ produced less acetate (decreased by 41.6%) than that at 55℃, obtaining 0.93 g/L butanol and 3.09 g/L ethanol from ~ 12 g/L sucrose in 50 ml bottles with a total biofuel yield of 0.33 g/g sucrose and butanol/total solvent ratio of 0.23 (w/w) (Additional file 1: Fig. S4).

All in all, our findings provided valuable insights into the possibility of butanol production by the genus *Thermoanaerobacterium*. As butanol titers were similar in all the four strains P8SB1–4, possibly it was limited by the reaction kinetics of the related pathways (e.g., thermodynamic equilibrium limit of the pathway for butanol production) in flask-scale [48], which could be researched for future work. In addition, butanol toxicities, imbalance of redox cofactors and implacable branch-pathways were major obstacles to achieve higher production in *Thermoanaerobacterium* [41, 49, 50].

### Molasses pre-treatment and *argR* deletion for enhanced molasses utilization

Molasses, known as sucrose-rich biomass derived from sugar production, can serve as cheap feedstock for biofuels production. Here, bioethanol production by ethanologenic mutants with unpretreated and pretreated molasses was first performed. The gene *rex* was deleted in strain P8S07 for higher ethanol production resulting in an 23% increase (strain P8S09) under sucrose in 50 mL serum bottles (Fig. 2B). However, under ~ 50 g/L untreated molasses, the growth of the mutants was obviously inhibited with a 1-day lag period in serum bottles due to the inhibitors in molasses, such as suspended colloids, metal ions and 5-hydroxymethylfurfural [11].

Several pre-treatment techniques for molasses were reported, including physical, chemical, physicochemical, and biological ones [3, 13]. For example, pretreatment with sulfuric acid [7, 51] and activated carbon [13] could remove most amounts of metal ions and other potential fermentation inhibitors from cane molasses. In addition, there is great potential to reduce pretreatment costs by developing robust strains with greater tolerance to inhibitors. As inhibitor tolerance of the microorganisms are involving multiple genes and several complicated regulation mechanisms [52–54], many researches recently focused on editing global regulators for enhanced stress resistance. For example, expression of global regulator *IrrE* from *Deinococcus radiodurans* enhanced furfural tolerance and exhibit stronger ROS scavenging ability of *Saccharomyces cerevisiae* [52]. The engineered *E. coli* with expression of *IrrE* showed enhancement of high salt resistance and could use seawater for succinic acid production [53]. In our previous study, deletion of the arginine repressor (ArgR, V518_1864), a global transcriptional regulator in *T. aotearoense* SCUT27, could significantly improve the expression of some chaperones, accounting for good abilities of anti-inhibitors and sugar utilization from cheap substrate hydrolysates [55].

**Fig. 4:**
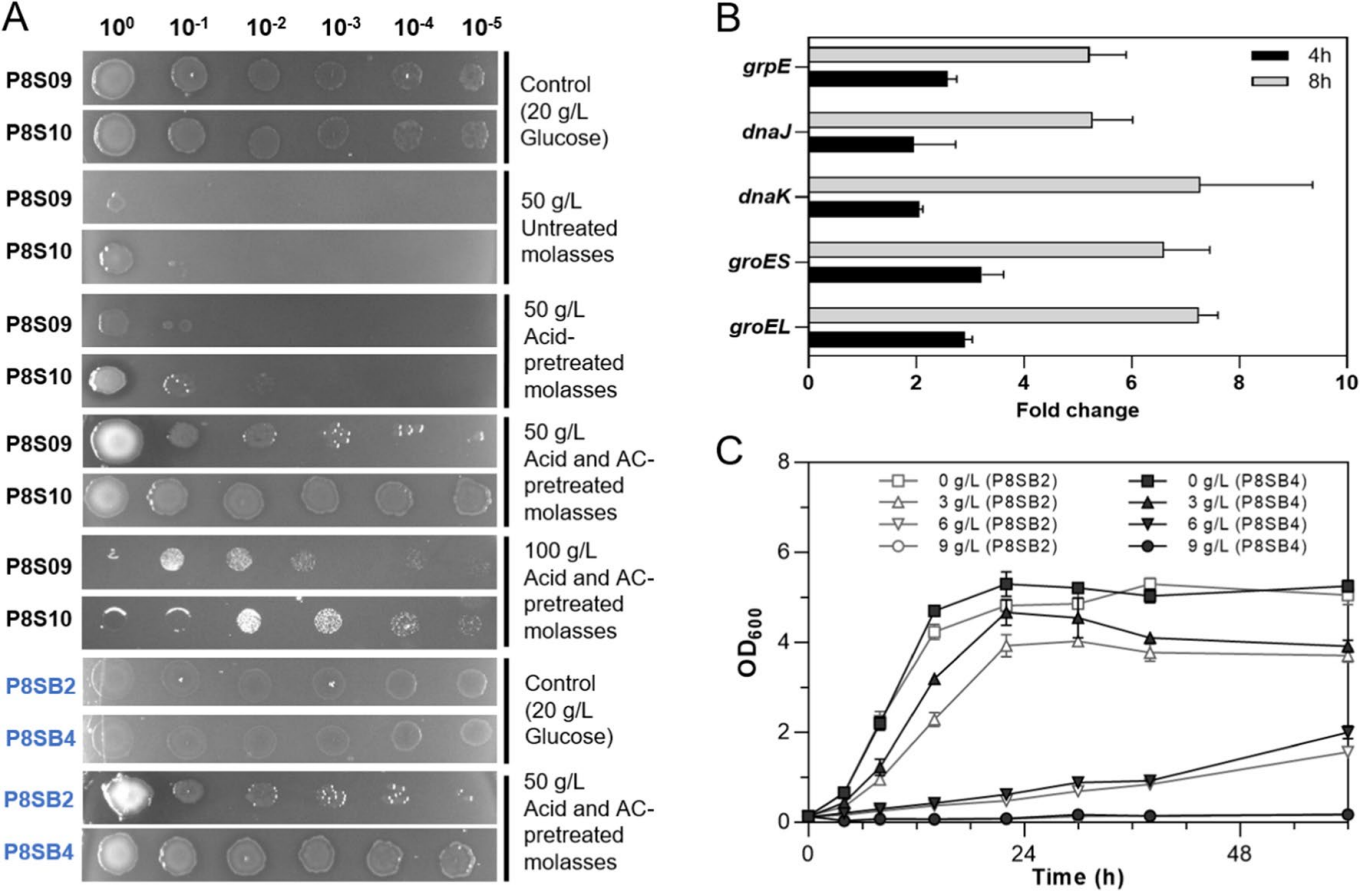
Effect of the pretreatment and genetic modification on the growth of the engineered strains. **A** Spot assays of the strains P8S09, P8S10, P8SB2 and P8SB4 on the plates using untreated, acid- or activated carbon (AC)-pretreated molasses. **B** Transcriptional level of chaperonins-related genes in strain P8S10 compared to that of strain P8S09 using molasses (with 16S rDNA as the internal reference). **C** Butanol tolerance test of the strains P8SB2 and P8SB4 with 0, 3, 6 and 9 g/L butanol, respectively

For lowering the toxicity of molasses conveniently, combination of acid and activated carbon (AC)-detoxification was applied and showed obviously positive effect on the tolerance of strains against the inhibitors in molasses, compared to the treatment with acid only (Fig. 4A). Furthermore, as *argR* knocked out, the resultant strain P8S10 grew better on molasses plates compared to strain P8S09, possibly due to the enhancement expression of GroEL–GroES chaperonin and DnaK–DnaJ–GrpE system (Fig. 4B), which could strengthen the bacterial abilities to scavenge reactive oxygen species (ROS) and resist the stress of various molasses-derived inhibitors [56]. It was also reported that co-expression of GroES, GroEL, DnaK, DnaJ and GrpE significantly upregulated the sucrose phosphorylase soluble expression and activities [29]. Similarly, strain P8SB4 with *argR* deletion based on strain P8SB2, showed better tolerance to inhibitors and butanol (Fig. 4A, C).

### Production of ethanol or co-production with butanol from molasses in fed-batch bioreactor cultivation

**Fig. 5:**
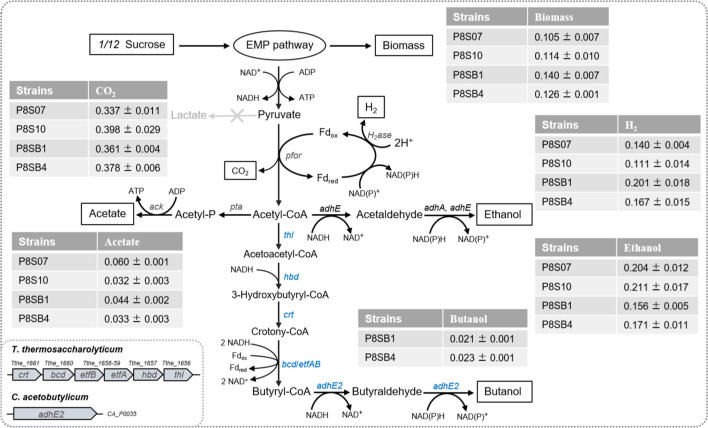
Fermentative pathway and carbon distribution of the strains P8S07, P8S10, P8SB1 and P8SB4 from sucrose. The genes in blue font were introduced from *T. thermosaccharolyticum* G3-1 or *C. acetobutylicum* to strain SCUT27 for butanol production. Abbreviation: *pfor*, pyruvate/ferredoxin oxidoreductase; *pta*, phosphate acetyltransferase; *ack*, acetate kinase. Numbers in tables represent the molar yield of end product based on per mole of carbon

Although biofuel production of the engineered strain has been greatly improved, it is necessary to see whether the carbon in the substrate is used as we wanted (whether the carbon flux to ethanol is more, and how much flux goes to butanol, etc.) after *rex* and *argR* deletion. Thus, the carbon distribution analysis of the strains P8S07, P8SB1, and their Δ*rex*/Δ*argR* mutants (P8S10 and P8SB4) from sucrose was performed (Fig. 5). The results suggested that knocking out *rex* and *argR* distributed more carbon flux to ethanol and CO_2_, while the production of acetate and hydrogen was reduced. It was speculated that the enhanced flux from pyruvate to acetyl-CoA with less H_2_ production supplied more redox cofactors, such as NAD(P)H to alcohols synthesis, and the reduced proportion of acetate gave more acetyl-CoA for alcohols production (Fig. 5). In fed-batch cultivation, the reduced acetate production was also beneficial for the growth of *Thermoanaerobacterium* [14].

**Fig. 6:**
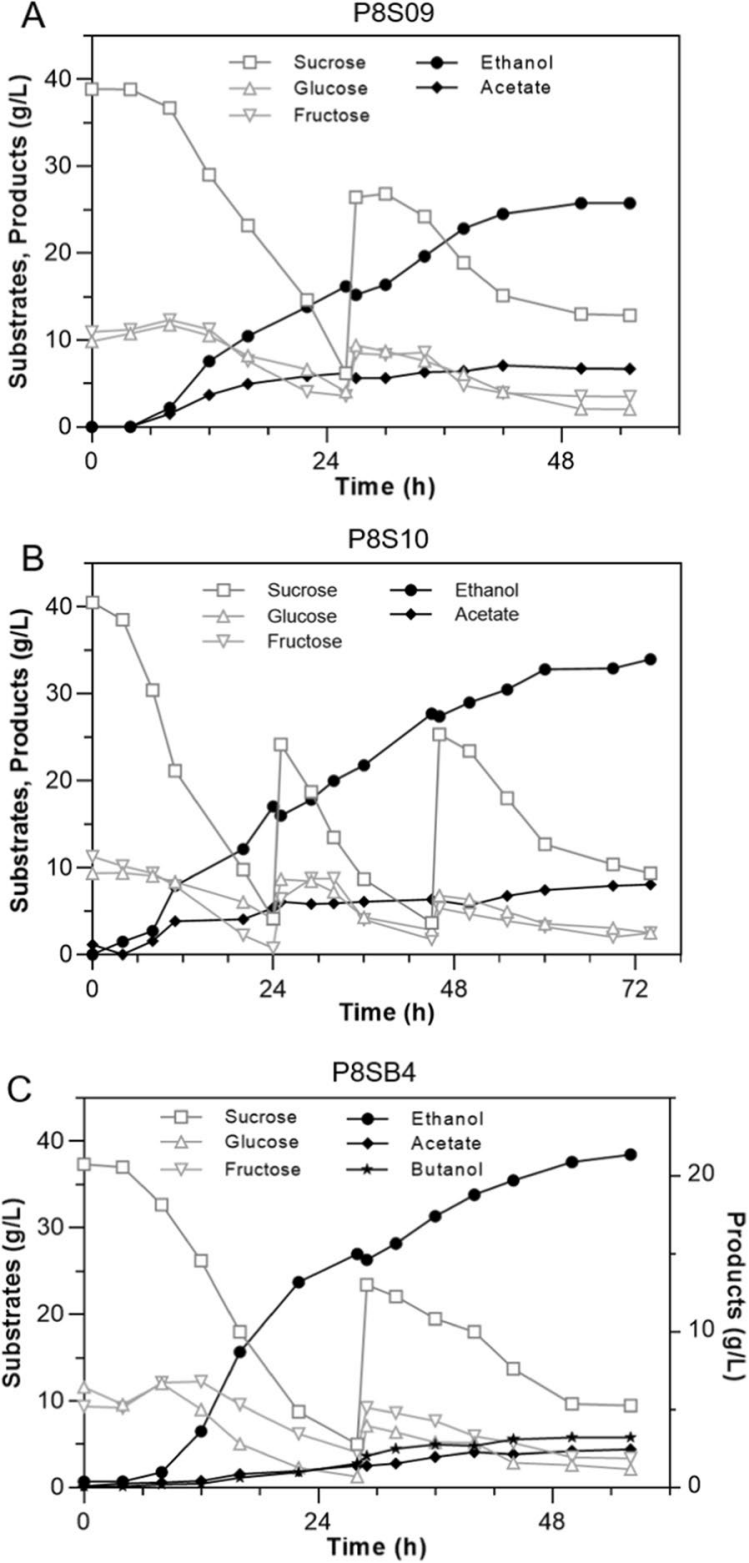
Molasses fermentation results of the engineered strains in 5 L bioreactor. Fed-batch fermentation kinetics of the strains P8S09 (**A**) and P8S10 (**B**) at pH 5.5 and 55 ℃ in 5 L bioreactor. **C** Fed-batch fermentation kinetics of the strain P8SB4 at pH 5.5 and 50 ℃ in 5 L bioreactor

In 5 L bioreactor, the final production of ethanol was up to 33.94 g/L with the yield of 0.39 g/g sugars by the engineered strain P8S10 from acid and AC-pretreated molasses after three times feeding, while the growth of strain P8S09 showed stagnated at about 42 h (two times feeding) (Fig. 6A, B). The results showed that sulfuric acid and AC co-pretreated method with the genetic modification (ArgR deletion) could significantly enhance the conversion of sugars in molasses to ethanol. Our work highlights new potential strategies for releasing constraints in adaptation to diverse environmental challenges. The butanol fermentation of strain P8SB4 was also performed in 5 L bioreactor with the same substrates and parameters except temperature at 50 °C. As shown in Fig. 6C, co-production of 21.36 g/L ethanol and 3.22 g/L butanol was obtained by P8SB4 with the yield of 0.38 and 0.06 g/g, respectively, after only feeding one time. The bacterial growth retardation after 48 h was possibly due to the inhibitory effect of butanol when the concentration was above 3 g/L (Fig. 4C). All in all, the titer of butanol here was at a high level among the reported *Thermoanaerobacterium* as well as the heterologous butanol-producing strains, such as yeast [57].

### Comparison to other studies and application prospect of *T. aotearoense* SCUT27

The biofuels titer and yield of the engineered strains with the sucrose utilization in this study were compared with those in other thermophilic microorganisms in Table 1. To date, few thermophiles (none of the genera *Thermoanaerobacterium* or *Thermoanaerobacter*) engineered for sucrose metabolism has been reported. The bioethanol yield of the mutant P8S10 here was up to 0.39 g/g sugars from molasses and the butanol production by P8SB4 was up to 3.22 g/L, which is comparable and even higher than previous studies (Table 1). However, the biofuel titer or yield (especially butanol) of the engineered *T. aotearoense* strains still has a big gap compared with that of mesophiles, such as *C. acetobutylicum* [42]. Some thermotolerant yeasts were used for high-temperature ethanol production, e.g., *Kluyveromyces marxianus* could ferment at 42 ℃ with high bioethanol production and yield comparable to the engineered *T. aotearoense* strains (Table 1). However, few yeasts could ferment well at temperatures above 50 ℃, and the butanol titer produced by yeasts was quite low [58, 59].

**Table 1:** Fermentation parameters of thermophiles for ethanol or butanol production

Strain	Genotype or phenotype	Substrate	Products	Fermentation mode	Titer (g/L)	Yield (g/g)	References
*Saccharomyces cerevisiae*	*Thermotolerant mutant* (40 °C)	molasses	Ethanol	5 L tank	72.40	0.36	[65]
*Kluyveromyces marxianus*	Wild type (42 °C)	Wheat straw	Ethanol	Fed-Batch	10.70–21.10	0.36–0.38	[66]
*P. kudriavzevii* DSA3.2	45 °C	Rice straw	Ethanol	SSF	10.53	0.43	[67]
*T. aotearoense strain* P8S10	Δ*ldh/*Δ*argR::scrB(G3-1)-scrA(CA)/*Δ*rex*	Cane molasses	Ethanol	Fed-Batch	33.94	0.39	This study
*T. thermosaccharolyticum DSM 571*	Overexpression of native *thl*, *hbd*, *crt*, *bcd*, *etfA*, and *etfB*	Cellobiose	Butanol	Batch	0.38	0.04	[40]
*T. saccharolyticum* JW/SL-YS485	Δ*ldh,* integration of butanol synthetic way	Xylose	Butanol	Batch	1.05	0.10	[41]
*Thermoanaerobacterium sp. M5*	Wild type	Xylan (+ FeCl_2_)	Butanol	Batch	1.17	0.04	[15]
*T. thermosaccharolyticum* TG57	Wild type (with no ethanol or butyrate byproducts)	Cellulose	Butanol	Batch	1.93	0.20	[68]
		Xylan	Butanol	Batch	3.63	0.23	
*T. aotearoense* strain P8SB2	Δ*ldh::adhE2-hbd-thl*/Δ*rex::crt-bcd-etfAB*/Δ*tdk::scrBA*	Sucrose	Butanol	Serum bottle	0.93	0.08	This study
			Ethanol		3.09	0.25	
*T. aotearoense* strain P8SB4	Δ*ldh*/Δ*argR::adhE2-hbd-thl*/ Δ*rex::crt-bcd-etfAB*/Δ*tdk::scrBA*	Molasses	Butanol	Fed-batch	3.22	0.06	This study
			Ethanol		21.36	0.38	

For future work, the expression level of the related enzymes in *T. aotearoense* SCUT27could be optimized by modification of strong promoters and RBS. In addition, fructose metabolism might be accelerated by overexpression of fructose metabolic genes or deletion of the potential transcriptional repressor [60]. As *T. aotearoense* SCUT27 has the great ability to utilize pentose [23, 24, 61], the waste of sugar refinery molasses and sugarcane bagasse hydrolysate (containing sucrose, glucose, fructose, xylose, arabinose and so on) could be co-utilized for biofuels production. Furthermore, co-cultured with other cellulolytic strains, such as *C. thermocellum*, *T. aotearoense* SCUT27 may make full use of molasses, bagasse and straw to achieve the maximum benefit. Low-cost nitrogen source, such as corn steep liquid [13], could also be considered for the biofuel synthesis with molasses together to further decrease the production cost.

## Conclusions

This study demonstrated the potential application of *T. aotearoense* SCUT27 for cost-effective biofuels production from sucrose-based feedstocks by expression of sucrose utilization and butanol synthetic pathways from *T. thermosaccharolyticum* G3-1 and *C. acetobutylicum* ATCC824. The mutant P8S10 with *rex* and *argR* deletion showed enhanced inhibitors-tolerance and produced ~ 34 g/L ethanol using molasses with a yield of 0.39 g/g sugars in 5 L tank. When overexpressing the pathway of butanol production (strain P8SB4), 3.22 g/L butanol was obtained with an alcohol yield of 0.44 g/g sugars. These strategies were feasible in thermophiles for improving stress tolerances and biofuels production with low cost molasses.

## Methods

### Organism and culture conditions

The strains used in this study are listed in Table 2. *T. thermosaccharolyticum* G3-1, *T. aotearoense* SCUT27 and the mutants were cultivated with modified MTC [20]. The solid MTC plates (2% agar, w/v) with 5 g/L sucrose were used for screening strains with the capacity of sucrose metabolism and ScrB activity test. The medium with 20 g/L sucrose or glucose was used for batch fermentation in 100-mL serum bottles at 55℃ and 150 rpm, anaerobically, and solid MTC medium containing 50 μg/mL kanamycin, 5 μg/mL thiamphenicol or 50 mg/mL 5-fluoro-2′-deoxyuridine (FUDR) was used for selection of transformants. *E. coli* DH5α used for plasmid construction was grown and selected at 37 °C aerobically with Luria–Bertani (LB) broth or plates containing 50 mg/mL kanamycin or 50 mg/mL chloramphenicol.

**Table 2:** Strains used in this study

Strain	Description	Source
SCUT27	Wild type of *T. aotearoense* SCUT27 (GDMCC 60765)	[64]
G3-1	Wild type of *T. thermosaccharolyticum* G3-1	This study
P8	Strain SCUT27/Δ*ldh::Erm*	[64]
P8S01	Δ*ldh::scrBAK(CA)::kanR*	This study
P8S02	Δ*ldh::scrBA(G3-1)::kanR*	This study
P8S03	Δ*ldh::scrB(G3-1)::kanR*	This study
P8S04	Δ*ldh::Tthe_1921-27::kanR*	This study
P8S05	*Δldh::Tthe_1923::kanR*	This study
P8S06	Δ*ldh::Tthe_1921::kanR*	This study
P8S07	Δ*ldh::scrB(G3-1)-scrA(CA)::kanR*	This study
P8S08	Δ*ldh::scrB(G3-1)-scrAK(CA)-scrK(G3-1)::kanR*	This study
P8S09	Δ*ldh::scrB(G3-1)-scrA(CA)::kanR*/Δ*rex::CAT*	This study
P8S10	Δ*ldh*/Δ*argR::scrB(G3-1)-scrA(CA)::kanR*/Δ*rex::CAT*	This study
P8S11	Δ*ldh::sp1-scrB(G3-1)::kanR*	This study
P8S12	Δ*ldh::sp2-scrB(G3-1)::kanR*	This study
P8S13	Δ*ldh::sp3*-*scrB(G3-1)::kanR*	This study
P8S14	Δ*ldh::sp4*-*scrB(G3-1)::kanR*	This study
P8S15	Δ*ldh::scrB(G3-1)-scrA(CA)::kanR::pIKM1_sp4*-*scrB(G3-1)::CAT*	This study
P8SB1	Δ*ldh::adhE2-crt-bcd-etfAB-hbd-thl::kanR*/Δ*tdk::scrB(G3-1)-scrA(CA)*	This study
P8SB2	Δ*ldh::adhE2-hbd-thl::kanR*/Δ*rex::crt-bcd-etfAB::CAT* /Δ*tdk::scrB(G3-1)-scrA(CA)*	This study
P8SB3	Δ*ldh*/Δ*rex::crt-bcd-etfAB::CAT*/Δ*adhE::adhE2-hbd-thl::kanR*/Δ*tdk::scrB(G3-1)-scrA(CA)*	This study
P8SB4	Δ*ldh*/Δ*rex::crt-bcd-etfAB::CAT*/Δ*argR::adhE2-hbd-thl::kanR*/Δ*tdk::scrB(G3-1)-scrA(CA)*	This study

### Isolation and identification of the strain

To isolate the thermophiles which could utilize sucrose, 1 g sludge from a spring in Yunnan was dissolved in 5 mL sterile water and the supernatant was transferred to the MTC medium for 2 days of cultivation (55 °C, 150 rpm). After several repeated enrichment processes, the broth dilutions were transferred on solid MTC plates with a gradient of concentration and cultured for 2–5 days at 55 ℃, anaerobically. The single bacterial colonies were transferred to fresh MTC liquid medium and the procedure of isolation was repeated at least three times to ensure the purity of colonies.

Genomic DNA was extracted by Bacterial DNA Kit (Omega, USA), and was used as the template for PCR amplification of 16S rDNA (universal primers: AGAGTTTGATCCTGGCTCAG and ACGGTTACCTTGTTACGACTT) and other genes, which then was sequenced (Sangon Co., Shanghai, China) and aligned using BLAST algorithm in NCBI nucleotide database. The phylogenetic dendrogram was constructed using the MEGA-X program with the neighbor-joining algorithm [62].

### Plasmid construction and transformations by electroporation

The plasmids and oligonucleotides used are listed in Additional file 1: Tables S4 and S5, respectively. Fragments were obtained by PCR using KOD DNA polymerase (Toyobo Co., Ltd.). Plasmids for editing were constructed by Gibson assembly with a ClonExpress MultiS one step cloning kit (Vazyme, Nanjing, China). The recombinant plasmids were transformed into *E. coli* DH5α cells (Weidi, Shanghai, China) for amplification and confirmed by colony PCR and DNA sequencing (Sangon, Shanghai, China). Plasmid isolation and purification were performed using TIANGEN kits. Sucrose catabolism genes were obtained by PCR amplification from the genomic DNA of *C. acetobutylicum* ATCC 824 and *T. thermosaccharolyticum* G3-1. The strong exogenetic promoter P_*cat1*_ [24] and native promoter P_*adhE*_, P_*ldh*_ or P_*crt*_ were used to express the genes of sucrose metabolism and butanol synthetic pathway.

Plasmids were transformed into *T. aotearoense* SCUT27 when OD_600_ reached ~ 0.8 as described previously [19, 20]. The colonies growing on selective plates were identified by colony PCR and then sequencing.

### ScrB hydrolase activity test

To measure the intracellular and extracellular sucrose hydrolysis activity of the engineered strains, cells were taken from the mid-exponential growth phase of the culture and centrifugated for 3 min at 8000 rpm. The supernatant was used for extracellular ScrB activity test and bacteria lysis solution with the same number of cells in PBS buffer (the same density before sample treated) was used for intracellular ScrB activity test.

Sucrose plate assay was applied for ScrB hydrolase activity test in this study through observing the difference in color development of iodine solution between reducing sugar and non-reducing sugar. The supernatant of the broth or cell lysis solution was spotted (5 μL) on the plates with the oxford cups, and incubated at 55 °C for 2 h. Then, 1 mL iodine solution (1 g iodine and 2 g potassium iodide dissolved in 300 mL H_2_O) was spread on the plate, and the hydrolysis circles were observed after 15 min. The enzymatic activity index (EI) was based on the size (diameter) of hydrolysis zone. In addition, the concentrations of glucose and fructose in medium containing 10 g/L sucrose with 10% of the supernatant or cell lysates of different mutants’ culture were measured after 2 h of incubation at 55 °C for identifying the ScrB location and hydrolase activity.

### Pretreatment of molasses and fermentation kinetics

The cane molasses from Bohua Food Co., Ltd (Guangxi, China) contained about 33.4% (w/w) sucrose, 9.5% (w/w) converted sugars (glucose and fructose), 7.3% (w/w) ash, 3.6% (w/w) salt, 2.9% (w/w) crude protein, 0.3% (w/w) crude fat, and 7.9% (w/w) metal ions, such as calcium, potassium, sodium, copper, etc. For acid treatment, the molasses solution was adjusted to pH 3.5 with 5 M H_2_SO_4_, and heated at 60 °C for 2 h. After centrifugation at 8,000 g for 15 min, the supernatant was adjusted to pH 6.0 with 10 M NaOH [51]. Pretreatment with 2% (w/v) activated charcoal was described by [13]. The pretreated molasses was finally diluted with water to obtain the sugars concentration about 60 g/L for initial fed-batch fermentation and 200 g/L for feeding.

Fed-batch fermentation kinetic of pretreated cane molasses were performed in 5-L bioreactor at 150 rpm and 55 °C, with pH controlled at 5.5 using ammonium hydroxide (30%, v/v). The bioreactor was sparged with nitrogen to achieved anaerobic condition and inculcated with 10% (v/v) seed culture. Samples were taken at regular time intervals to detect OD_600_ and centrifuged at 12,000 rpm for 10 min to gain supernatant for analyzing. All the experiments were performed at least twice.

### Spot assay and real-time quantitative PCR (qPCR)

The mutants of *T. aotearoense* SCUT27 were cultured with 10 g/L glucose for activating before spotting on the plates. When OD_600_ was 2.0, 1 OD_600_/mL cells was prepared after washing strains and re-suspending them with sterile water. Followed by a series of gradient dilution, 5 μL diluted cells were pipetted on various MTC plates with untreated, acid- or activated carbon-pretreated molasses, respectively, and cultured for 24 h at 55 ℃ anaerobically. In addition, the samples of activated strains were taken at 4 h and 8 h after inoculation for RNA extraction and qPCR analyzing with primers presented in Additional file 1: Table S5.

### Analytical methods

The amino acid sequence identity of sucrose metabolism systems among *T. thermosaccharolyticum* G3-1, *T. aotearoense* SCUT27 and *C. acetobutylicum* ATCC 824 was performed on Protein Blast (https://blast.ncbi.nlm.nih.gov/Blast.cgi). The signal peptides were chosen in the genome from NCBI, marked and scored by SignalP-5.0 [35] as shown in Additional file 1: Table S4. The OD_600_ was detected with Spectrophotometer (PERSEE T6, Beijing, China). Sugars (sucrose, glucose and fructose), acetic acid, ethanol and butanol were determined by HPLC (Waters, Milford, USA) with a CarboPac H^+^ column (Dikma, Beijing, China) at 30 °C and a refractive index detector (Waters, Milford, USA) [11]. In addition, 1.0 mM H_2_SO_4_ solution at 0.6 mL/min was used as the mobile phase.

Analysis of the carbon metabolic distribution was performed with MTC medium under 20 g/L sucrose. The gas in serum bottles was measured by gas chromatography (Fuli 9790, China) and calculated as description in [19, 63]. The relationship between OD_600_ and cell dry weight (CDW) was 0.46 g/L/OD_600_ and the cell composition was estimated using the general empirical formula CH_2_N_0.25_O_0.5_, according to previous study [64]. The carbon in yeast extract was not calculated in carbon metabolic distribution. All substances (sugar consumed, biomass and products) were first calculated to their quantity concentration (*n*/*V*), and 30 (CH2O) was used for sugar as molar mass (M). Then, *n*/*V* value of each substance was divided by the *n*/*V* value of the sugar consumed, respectively. The resultant number (*x*) was estimated as the quantity of the substance which was converted from one unit quantity of the sugar (CH2O), and the sum of all *x* multiplying by the carbon number of their corresponding substances should be equal to one unit in theory due to the carbon conservation, as the following equations:

1 $${1}\cdot{\text{CH}}_{{2}} {\text{O }} = \,x_{{1}} \cdot{\text{CH}}_{{2}} {\text{N}}_{{0.{25}}} {\text{O}}_{{0.{5}}} \left( {{\text{biomass}}} \right) \, + x_{{2}} \cdot{\text{C}}_{{2}} {\text{H}}_{{6}} {\text{O }}\left( {{\text{ethanol}}} \right) \, + x_{{3}} \cdot{\text{C}}_{{4}} {\text{H}}_{{{1}0}} {\text{O }}\left( {{\text{butanol}}} \right) \, + x_{{4}} \cdot{\text{C}}_{{2}} {\text{H}}_{{4}} {\text{O}}_{{2}} \left( {{\text{acetate}}} \right) \, + x_{{5}} \cdot{\text{CO}}_{{2}} + x_{{6}} \cdot{\text{H}}_{{2}}$$

2 $${1 } = x_{{1}} + x_{{2}} \cdot{2} + x_{{3}} \cdot{4 } + x_{{4}} \cdot{2 } + x_{{5}} .$$

### Supplementary Information

**Additional file 1: Figure S1.** Traits of *T. thermosaccharolyticum* G3-1 compared with *T. aotearoense* SCUT27. (A) The neighbor-joining phylogenetic tree of some thermophilic anaerobacteria or common clostridia was constructed based on the 16 s rDNA sequence, showing the position of *T. thermosaccharolyticum* G3-1. (B) The growth rate and end products of *T. thermosaccharolyticum* G3-1 and *T. aotearoense* SCUT27 under different sugars. **Table S1**. Plasmids used in this study. **Table S2**. Primers used for gene amplification and qPCR in this study. **Table S3.** Sequence of sucrose metabolism genes in *T. thermosaccharolyticum* G3-1. **Table S4.** Sequence of the signal peptides used in this study. **Table S5.** Hydrolysis circle size for ScrB activity test.

## Data Availability

Data will be made available from the corresponding author on reasonable request.

## Abbreviations

ScrB: Sucrose-6-phosphate hydrolase
PTS: Phosphoenolpyruvate-dependent phosphotransferase system
ScrA: Sucrose-specific PTS transporter
ScrK: Fructokinase
Rex: Redox-sensing transcriptional repressor
ArgR: Arginine repressor
CCR: Carbon catabolite repression
Ldh: Lactate dehydrogenase
MTC: Medium for thermophilic clostridia
FUDR: 5-Fluoro-2′-deoxyuridine
PMS: Sucrose permease system
AC: Activated carbon
Thl: Thiolase
Hbd: β-Hydroxybutyryl CoA dehydrogenase
Crt: Crotonase
Bcd: Butyryl CoA dehydrogenase
EtfAB: Electron transfer flavoproteins subunit A and B
Adh: Alcohol dehydrogenase
