# Supplementary material for: Metabolic engineering of Thermoanaerobacterium aotearoense strain SCUT27 for biofuels production from sucrose and molasses
Source: Biotechnol Biofuels Bioprod. 2023 Oct 21;16:155. doi: 10.1186/s13068-023-02402-3 (PMC10589968; doi:10.1186/s13068-023-02402-3)
Supplement: Supplementary file 1 — Additional file 1: Figure S1. Traits of T. thermosaccharolyticum G3-1 compared with T. aotearoense SCUT27. (A) The neighbor-joining phylogenetic tree of some thermophilic anaerobacteria or common clostridia was constructed based on the 16 s rDNA sequence, showing the position of T. thermosaccharolyticum G3-1. (B) The growth rate and end products of T. thermosaccharolyticum G3-1 and T. aotearoense SCUT27 under different sugars. Table S1. Plasmids used in this study. Table S2. Primers used for gene amplification and qPCR in this study. Table S3. Sequence of sucrose metabolism genes in T. thermosaccharolyticum G3-1. Table S4. Sequence of the signal peptides used in this study. Table S5. Hydrolysis circle size for ScrB activity test. [file 13068_2023_2402_MOESM1_ESM.docx]

**Additional File 1**

**Figure S1.** **The traits of *T. thermosaccharolyticum* G3-1 compared with** ***T.*** ***aotearoense* SCUT27.** (A) The neighbor-joining phylogenetic tree of some thermophilic anaerobacteria or common clostridia was constructed based on the 16s rDNA sequence, showing the position of strain G3-1. (B) The growth rate and end products of strain G3-1 and strain SCUT27 under different sugars.


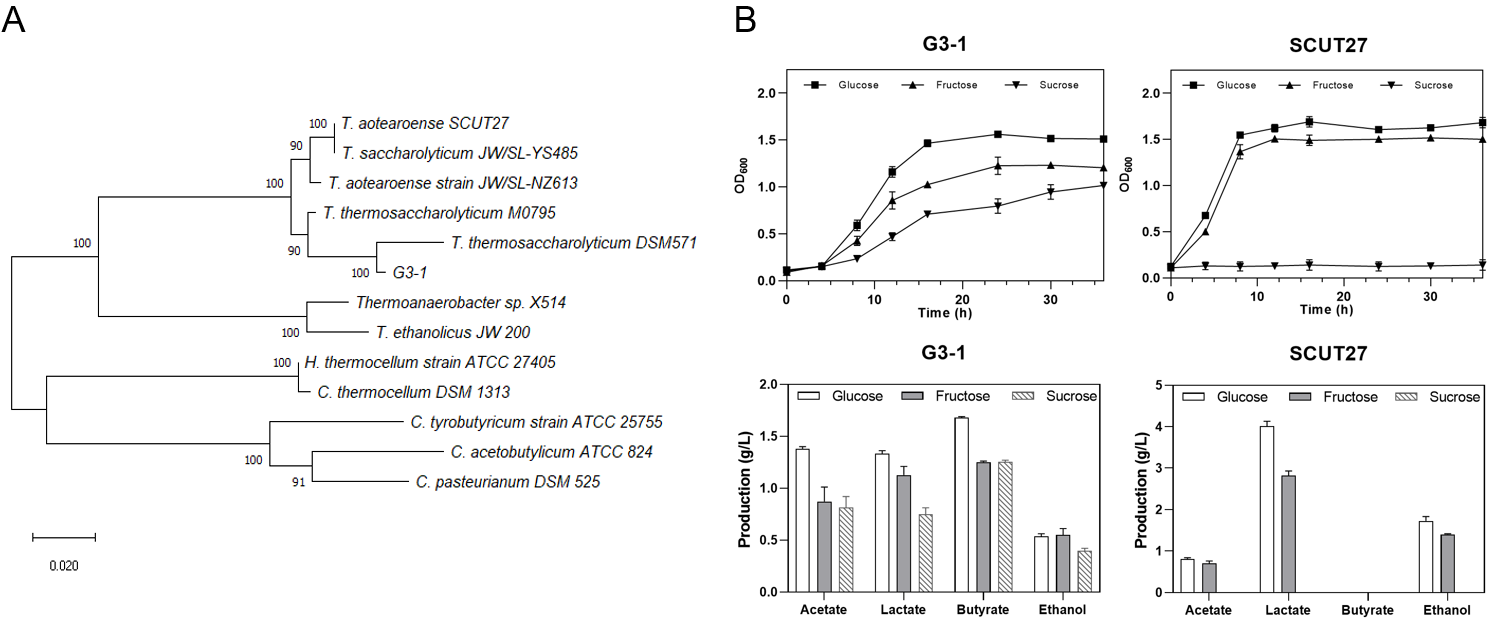


**Figure S2. Rex regulation for gene *crt*.** (A) The potential rex binding site in the promoter region of *crt*. (B) The relative expression level of *crt* in strains P8S11 and P8S12 (*, *P* ≤ 0.05; **, *P* ≤ 0.01).


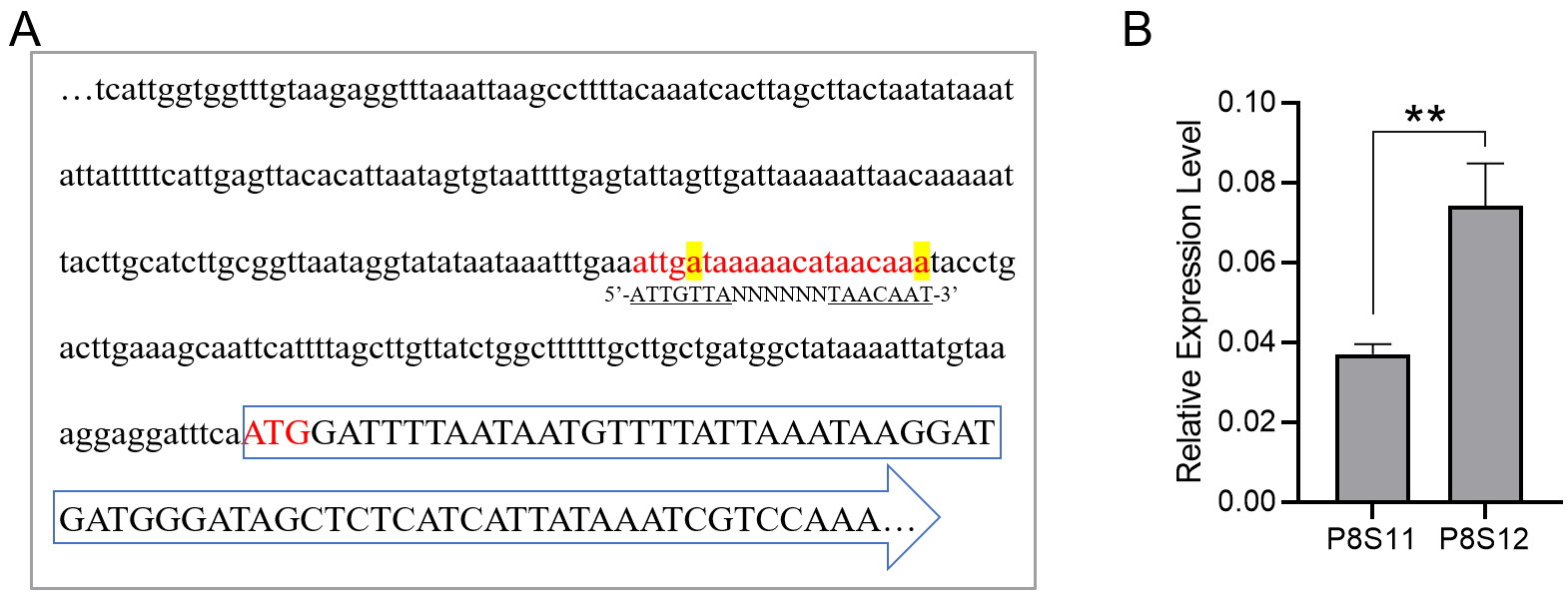


**Figure S3. The effect of *adhE* knockdown on growth of *T.* *aotearoense* SCUT27.**


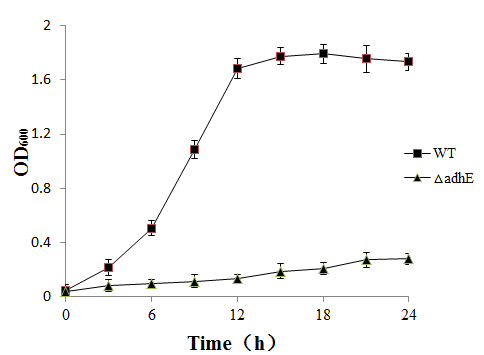


**Figure S4. The fermentation profiles of the strain P8SB2 under different temperatures.**


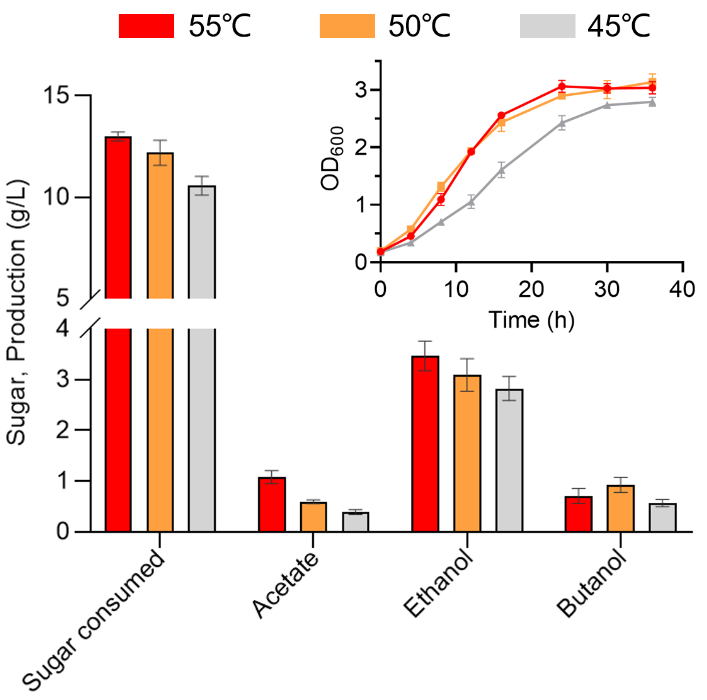


**Table S1**

The sequence of sucrose metabolism genes in *T. thermosaccharolyticum* G3-1.

| ***scrA*** |
| --- |
| ATGAAGTATGAAAAATTAGCCAAAGACATTATAAAAAATGTTGGCGGCAAAGAGAATGTAATTAGTTTAACCCACTGTATTACACGTCTTCGCTTTAAACTAAAGGACGAAAGCAAGGCAAATACAGAAACCCTTAAAAAAATGGATGGTATTGTTACCGTTGTAAAAAGTGGTGGACAATATCAGGTTGTTATAGGAAACCATGTACCAGATGTATATGCTGATGTTGTTGCTATAGGAGGTTTTCAGACAGAAGGTACAGAGAAAGCAGCATCAGGTGAAAAGAAAAGTTTATTTAACAGCTTTATTGATACTATTTCAGGTGTATTTGCACCAACATTAGGTGTGCTTGCTGCAACAGGTATGATAAAAGGCTTAAATGCATTACTTATATCAATGCATTTACTTACTGCGACTTCAGGAACTTATGAGATTTTAAATGCCATAGGAGACAGTTTTTTTTACTTCCTTCCTATTTTCCTCGGATTTACAGCTGCTAAAAAGTTTAATGCTAATCAGTTTATTGGCATGACGCTGGGTGCAATACTTGTTTATCCGACATTTACTAGTATAATGGCAGGGAAACCTTTGTACACATTATTTAATGGAACGGCAATTCAATCGCCTGTGTATTTAACTTTCTTAGGTATTCCTGTTATATTAATGAGTTATTCATCAAGCGTTATTCCTATTATTTTAACCGTATATGTAGGAGCTAAAATTGAGAAATTCTTTAAAAAAATAATACCTGATGTAGTTAAAACATTCTTGGTGCCGTTCTTTACACTTTTAGTAGTTGCTCCTTTAGCTTTAATAGTTATAGGACCTATAGCAACTTGGGCAGGTAAATTACTTGGCGCATTAACGATTGCTATATACAACTTAAGCCCGATCATTGCAGATTATTTATGGGTGCTTTTTGGCAAGTGTTTGTTATATTTGGGTTACACTGGGGATTTATACCAATAATGATCAACAATTTATCACTACTTCATTATGATACAATTGTTGCATTAACATTTGCAGCTTCTTTTGCTCAAACAGGTGTGGTATTAGCTGTTTTAGTTAAAACGAAGAATCCTAAATTAAGATCAATAGCAATACCGGCATTTGTTTCAGGGATTTTCGGTGTTACAGAACCAGCAATATATGGTGTTACATTGCCTCGTAAAAAACCGTTTATACTAAGTTGTATAGCAGCTGCAGTAGGCGGTGGTATTATGGGATTTATGGGAACTAAAATGTATATGCTTGGTGGACTTGGTATTTTTGGAATTCCAAGTTTTATTAGTCCAAAAGGAATTGACACTGGATTCTATGGTGTAATTATTTCCATAGTGGTAGGGTTTGTATTAGGTTTCATATTAATGCTCTTCAGTGGTCTTAAAGACGAAGAATCTAAAGAAGAAAATAAGGAGGTAACCAGTGGAGATATATTAATAAAGCAAGAAACTGTAGTTAGCCCATTAAAAGGGGAAGTCAAAGCCTTATCAGAAATAAAGGACGAGGCTTTTTCAAAGGGTGTACTAGGCAAAGGCGTTGCAATCGAACCAACAGAAGGCAGGATAGTTGCACCTGTAGATGGAACTGTAACAACTCTATTTCCAACTGGTCATGCTTTAGGAATTACAAGCGATAAGGGTACAGAAATTTTAATTCATATTGGTATGGATACAGTTCAATTGGAAGGCAAATATTTTCATGCTAAAGTAAAACAAGGTGATAATGTAAAGGCAGGTCAGGTACTGGTAGAATTCGACATTGATGCTATAAAGAAGGAAGGTTATTCTTTAACAACACCTATAGTGATTACAAATTCAAATAATTACTTGGATGTTATTGAAACAGATAAAAAGACTGTCAACTATAAAGATGATTTATTAACAGTGATGATCTAG  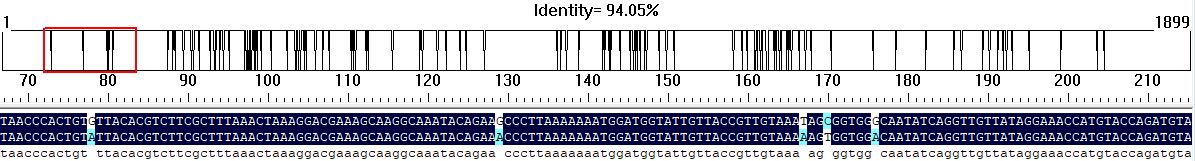 |
| ***scrB*** |
| ATGAACAAAATTGATGAAGCAAATGAATTTATAAAACAAAAAAAGCAAAAGGTAAATCCTAAGTACAGGCTAAAGTATCATTTGATGGGAGAATATGGGTGGATAAACGACCCAAATGGGTTCATACAGTATAAAGGAAATTACCATTTATTCTATCAACATTATCCATATGATGCTGTATGGGGACCAATGCATTGGGGGCATGCCATAAGCAAAGACTTGGTTAAGTGGTTTTACTTGCCTTTGGCTTTAGCACCTGAAGAAGATTATGATAGAGATGGGTGCTTTTCTGGAAGTGCTATCGAAAAAAACGGGAAATTATACCTTTTTTACACAGGACATATATACACTAAAAAAGAAAAAAACGATGATTACAAACAGGTTCAGAATATGGCCATATCAGCAGATGGAATTGCATTTGAAAAGTATGAAAAAAATCCGATAATAGATGTAACACAGATTCCAGATAAAGCTAGCAAGAAAGATTTTAGAGATCCAAGAATTTTTAAAATAGGTGATACCTATTATCTTTTAATTGGCTCTAATGATGAACACGGAATTGGACAGATTCTCATGTATAAGTCAATCGATTTAATAAAATGGGAATTTGTAAATATTCTTTTAAAGGGTAATGAAAATACAGGCATTAACTGGGAATGTCCCGATATAATTCGCTTTGAAGAAAAAGATATTTTATTAGTATCAGCACAATATATGAAGGCTAAAGGTGATGACTTCAAAAATACACATTCATCTATTTACTTTGTGGGCAGATTAGATATAGATAAAGGAAAATTTGAATATGAAAATTATTACTCAATTGATTATGGGTTTGATTTTTATGCACCACAAACTACCATAGATAAGAATGGAAAGATAGTAATGATAGCATGGATGAACATGTGGGAGACAGATTTGGTTACAAATCGTCTTGGTCATAATTGGGCTGGTGCTATGACGCTTCCTAGAGAAGTTATAAAAATAGGTGAGGAGATATACTTTAAGCCAATATCAGAGATAGTTAAGTATAGGAAAAATGAATACAGCTTGCAAGACATAGAATTAAATGGTGAAGTTAATCTTGAAACGAATGGAATATGTTATGAGATTGATGCTGAATTTGAATCGCTAGATGCAAAAGAATTTGGATTAAAAGTGAGAAAAGGTAAGAAGGAAGAGACTGTTTTGTCATATAACTGTCAAGAGAGTTTATTCATATTTAATCGCGACAGATCGGGCGTTGGGCCAAAAGGAGAAAGGAAGACACTGGTTAACTTAAATGAAGGAAGATTGAGGCTTAGGGTCTTTGTGGATGTGTCATCCGTGGAGGTTTTTATAAATGAAGGTGAAGAGGTAATGACTGGAAGGATATATCCAGACAGTGAATCGATTTATATTTCAATATTTTCTGTGGGCGAATGCAAGAAGTTCATTTATTATCCTTAA  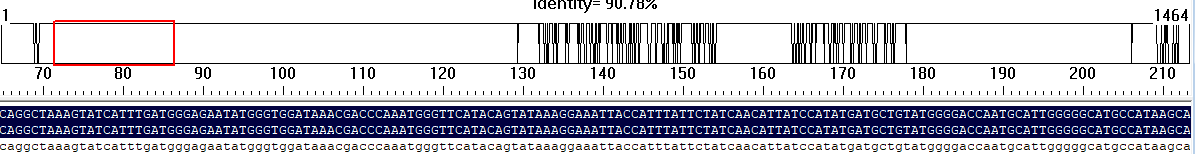 |

**Table S2**

Sequence of the signal peptides used in this study.

| sp1: from V518_2545 (endo-1,4-beta-mannosidase); Sec/SPII; expressed in P8S11 |
| --- |
| ATGAAAAAGTTTTGTATTCTTCTGATGTGCATTATCATTCTTATAAGTGGGTGCAAATTTAAT |
| sp2: from V518_0231 (periplasmic sugar-binding protein); Sec/SPII; expressed in P8S12 |
| ATGAAAACATTTAGGTTATGGCTTGTAGTTGCTTTGATGATTTTCGCAACTTTTGCATTTGCAGGTTGTGGCAACAGC |
| sp3: from V518_1453 (cell wall hydrolase Sleb); Sec/SPI; expressed in P8S13 |
| ATGTTAAAAAAATGTATTAAGGTAAAGCCTCTCATTGCTTCATTAGCAGCGGCTCTTTTATTTTCTCAGACGGCATTTGCGGCAACGTATACT |
| sp4: from V518_1647 (pepsY domain-containing protein); Sec/SPI; expressed in P8S14 |
| ATGAAAAAATTAATAGCTTCTGTATTTGCATTTATAATTTTGACAGGAATATTTAATGTTTATGCAGCGGAAACTTCA |

**Table S3**

The concentrations of glucose and fructose in 10 g/L sucrose medium (SM) with 10% of the supernatant or cell lysates of different mutants’ culture, and the hydrolysis circle size for ScrB activity test (with the cell lysates or the supernatant of the culture “*”).

| Strains | SM with supernatant | | SM with cell lysates | | Hydrolysis circle diameter on plates (mm) |
| --- | --- | --- | --- | --- | --- |
|  | Glucose (g/L) | Fructose (g/L) | Glucose (g/L) | Fructose (g/L) |  |
| P8 | 0.09 ± 0.06 | 0.07 ± 0.07 | 0.11 ± 0.11 | 0.06 ± 0.01 | 0.0 |
| P8S02 | 0.95 ± 0.18 | 1.01 ± 0.20 | 1.40 ± 0.37 | 1.88 ± 0.06 | 11.0 ± 1.08 |
| P8S03 | 1.11 ± 0.28 | 0.85 ± 0.29 | 2.08 ± 0.18 | 1.61 ± 0.23 | 10.3 ± 1.03 |
| P8S04 | 0.82 ± 0.21 | 1.01 ± 0.36 | 2.12 ± 0.30 | 2.06 ± 0.15 | 10.2 ± 1.03 |
| P8S07 | 1.12 ± 0.26 | 1.33 ± 0.19 | 1.98 ± 0.13 | 2.16 ± 0.20 | 13.2 ± 1.43/  13.5 ± 0.41* |
| P8S11 | 1.99 ± 0.14 | 1.84 ± 0.08 | 1.78 ± 0.16 | 2.02 ± 0.23 | 14.8 ± 0.62* |
| P8S12 | 1.87 ± 0.33 | 1.98 ± 0.31 | 2.22 ± 0.35 | 1.85 ± 0.17 | 16.2 ± 1.03* |
| P8S13 | 3.59 ± 0.02 | 3.35 ± 0.38 | 1.94 ± 0.21 | 1.80 ± 0.02 | 22.7 ± 0.62* |
| P8S14 | 3.91 ± 0.26 | 3.70 ± 0.27 | 1.74 ± 0.21 | 2.14 ± 0.18 | 23.0 ± 1.63* |

**Table S4**

Plasmids used in this study.

| Plasmid | Description | Sourse |
| --- | --- | --- |
| pBluescript II SK(+) | Suicide plasmid used for gene deletion and integration | Stratagene |
| pIKM1 | Original plasmid for cloning and expression with *AmpR* and *KanR* | (Mai et al., 1997) |
| pBlu-Δ*ldh* | pBluescript II SK(+)-homologous fragment of *ldh* /*KanR* | This study |
| pBlu-Δ*tdk* | pBluescript II SK(+)-homologous fragment of *tdk* | This study |
| pBlu-Δ*rex* | pBluescript II SK(+)-homologous fragment of *rex* /*CAT* | This study |
| pBlu-Δ*argR* | pBluescript II SK(+)-homologous fragment of *argR* /*KanR* | This study |
| pBlu-Δ*adhE* | pBluescript II SK(+)-homologous fragment of *adhE* /*KanR* | This study |
| pBlu-Δ*ldh*-S01 | pBlu-Δ*ldh* ::*scrBAK(CA)* /*KanR* | This study |
| pBlu-Δ*ldh*-S02 | pBlu-Δ*ldh* ::*scrBA(G3-1)* /*KanR* | This study |
| pBlu-Δ*ldh*-S03 | pBlu-Δ*ldh* ::*scrB(G3-1)* /*KanR* | This study |
| pBlu-Δ*ldh*-S04 | pBlu-Δ*ldh* ::*Tthe_1921-27* /*KanR* | This study |
| pBlu-Δ*ldh*-S05 | pBlu-Δ*ldh* ::*Tthe_1923* /*KanR* | This study |
| pBlu-Δ*ldh*-S06 | pBlu-Δ*ldh* ::*Tthe_1921* /*KanR* | This study |
| pBlu-Δ*ldh*-S07 | pBlu-Δ*ldh* ::*scrB(G3-1)-scrA(CA)* /*KanR* | This study |
| pBlu-Δ*ldh*-S08 | pBlu-Δ*ldh* ::*scrB(G3-1)-scrA(CA)-scrK(G3-1)*/*KanR* | This study |
| pBlu-Δ*ldh*-S11 | pBlu-Δ*ldh* ::*sp1-scrB(G3-1)*/*KanR* | This study |
| pBlu-Δ*ldh*-S12 | pBlu-Δ*ldh* ::*sp2-scrB(G3-1)*/*KanR* | This study |
| pBlu-Δ*ldh*-S13 | pBlu-Δ*ldh* ::*sp3-scrB(G3-1)*/*KanR* | This study |
| pBlu-Δ*ldh*-S14 | pBlu-Δ*ldh* ::*sp4-scrB(G3-1)*/*KanR* | This study |
| pIKM1-*sp4-scrB* | pIKM1-P*cat1*-*sp4-scrB(G3-1)-CAT* | This study |
| pBlu-Δ*argR*-S01 | pBlu-Δ*argR* ::*scrB(G3-1)-scrA(CA)*/*KanR* | This study |
| pBlu-Δ*tdk*-S01 | Δ*tdk::scrB(G3-1)-scrA(CA)* | This study |
| pBlu-Δ*ldh*-SB1 | Δ*ldh::adhE2-crt-bcd-etfAB-hbd-thl* /*KanR* | This study |
| pBlu-Δ*ldh*-SB2 | Δ*ldh::adhE2-P_cat1_-hbd-thl* /*KanR* | This study |
| pBlu-Δ*rex*-SB1 | Δ*rex::crt-bcd-etfAB::CAT* | This study |
| pBlu-Δ*adhE*-SB1 | Δ*adhE::adhE2-*P*_cat1_-hbd-thl* /*KanR* | This study |

**Table S5**

Primers used for gene amplification and qPCR in this study.

| **Primers** | **Sequence (5′ to 3′)** |
| --- | --- |
| **Vector construction** |  |
| *ldh*-H1-F | tccaccgcggtggcggccgctctagaCATGCGTCAATAGAAATTGAC |
| *ldh*-H1-R | GTCCTTATTGATTTGAAATATTATCT |
| *ldh*-H2-F | GTCATGGCAGATGTCATAAAAC |
| *ldh*-H2-R | actcactatagggcgaattgggtaccCATGCATCACAATCTTGTCTATG |
| *tdk*-H1-F | tccaccgcggtggcggccgctctagaCAACATGGAGCTGCACCT |
| *tdk*-H1-R | GTGGTCTTTAGGCCCATACA |
| *tdk*-H2-F | GTGTAGAAAGTGCCATGAAGTC |
| *tdk*-H2-R | actcactatagggcgaattgggtaccGATATGCCTGCGACGACA |
| *rex*-H1-F | tccaccgcggtggcggccgctctagaCAGTGAAAGATGGTTGCTTACT |
| *rex*-H1-R | ACAGGCATCGGCACTATC |
| *rex*-H2-F | TGACCTTGTCGTATCGCAT |
| *rex*-H2-R | actcactatagggcgaattgggtaccTGTGGCATTACGACAGCAC |
| *argR*-H1-F | tccaccgcggtggcggccgctctagaCAATGGACCGCAGGTAGG |
| *argR*-H1-R | CTCCTCTATAAGCTGTCTTTGG |
| *argR*-H2-F | AGCAGCACAGTGGCTAAAG |
| *argR*-H2-R | actcactatagggcgaattgggtaccCAAGGCATCAAACAGCTCG |
| *adhE*-H1-F | tccaccgcggtggcggccgctctagaGCCACTCATCCTCTCAAAGTG |
| *adhE*-H1-R | AATAAACACCTCCGTGTTAATTT |
| *adhE*-H2-F | CCCGAGAATGCCTCTCAC |
| *adhE*-H2-R | actcactatagggcgaattgggtaccGATGCAGTAAAGACTGGCG |
| *Pcat1-*F | AGAGCCCAAATCTTTGAAAAT |
| *Pcat1-*R | AAAAACCACCCTTTCATAAATT |
| *scrB(CA)-*F | ATGAATAGCTTGGAATTTATAAAGA |
| *scrB(CA)-*R | GTCTTATATTGCTTTATGCTGATAG |
| *scrAK(CA)-*F | AGGAGGGAAAATATATGGATTATA |
| *scrAK(CA)-*R | CTAATCACCTTCAACTTCACTTAAA |
| *scrB(G3-1)-*F | aatttatgaaagggtggtttttATGAACAAAATTGATGAAGCAAAT |
| *scrB(G3-1)-*R | tacatcgcctcctaataattcgTTAGTCGTAAGATTAATCTACGTCG |
| *scrA(G3-1)-*F | cgaattattaggaggcgatgtaATGAAGTATGAAAAATTAGCCAAAG |
| *scrA(G3-1)-*R | tttatgacatctgccatgacCTAGATCATCACTGTTAATAAATCATC |
| *Tthe_1921-*F | gaaaagaggaaggaaataataaATGGCGCTTAATAATAAAGTACAA |
| *Tthe_1921-*R | tacatcgcctcctaataattcgTTATACTAAATACTTTACTTCTTCACCG |
| *Tthe_1927-*F | gaaaagaggaaggaaataataaATGAAGAGAAAAAAACTATTATCAATG |
| *Tthe_1923-*F | gaaaagaggaaggaaataataaATGAAAAAAGCATGGTGGAAG |
| *Tthe_1923-*R | tacatcgcctcctaataattcgTTACACCTTTAAAAGATAAACTCTTGA |
| *scrA(CA)-*R | TTACTTTATTCCAATCTTTAAAATTTC |
| *scrK(G3-1)-*F | ATGTTTAATTTTAATGACAAGATTGT |
| *scrK(G3-1)-*R | tacatcgcctcctaataattcgTCACCTCAGTTTTTTTAAATCGT |
| *adhE2(CA)-*F | ATGAAAGTTACAAATCAAAAAGAACTA |
| *adhE2(CA)-*R | TACATCACCGACGAGCAAG |
| *crt(G3-1)-*F | TCATTGGTGGTTTGTAAGAGG |
| *etfAB(G3-1)-R* | TGTCAAACTGATCAGCTGCC |
| *hbd(G3-1)-*F | ATGCAAAAGATTTGTGTAATAGGT |
| *thl(G3-1)-*R | CTATCTTTCGACAACCATTGC |
| **qPCR** |  |
| *dnaK*-F | TGGGCAAAGAACCGCACAAAGG |
| *dnaK*-R | CGCCAAGTGTTTCAATGCCAAGAG |
| *dnaJ-*F | AGAGGACGTGGTGACGAATTTGTG |
| *dnaJ*-R | ACCGCTATCATTGCTCAGCTTGTC |
| *grpE*-F | TGACAGTGAAGGCAGCATAGGT |
| *grpE*-R | TCTGCTTTCAATCTCTGGGCCA |
| *groEL*-F | TGGTGATGGTACAACAACGGCTAC |
| *groEL*-R | ATCCTTCAACAGCAGCATCGACAG |
| *groES*-F | TGTATTGCCTGGCACTGCGAAAG |
| *groES*-R | TCCGACTTTTACTTCCAGCTCGAC |
| 16S rDNA-F | CTCGTGTCGTGAGATGTTGG |
| 16S rDNA-R | GCCTTCCTCCGTGTTATCC |
